# The Application of Biochar for CO_2_ Capture: Influence of Biochar Preparation and CO_2_ Capture Reactors

**DOI:** 10.1021/acs.iecr.3c00445

**Published:** 2023-06-29

**Authors:** Chen Zhang, Ying Ji, Chunchun Li, Yingrui Zhang, Shuzhuang Sun, Yikai Xu, Long Jiang, Chunfei Wu

**Affiliations:** † Institute of Refrigeration and Cryogenics, Zhejiang University, Hangzhou 310027, China; ‡ School of Chemistry and Chemical Engineering, Queens University Belfast, Belfast, U.K. BT7 1NN

## Abstract

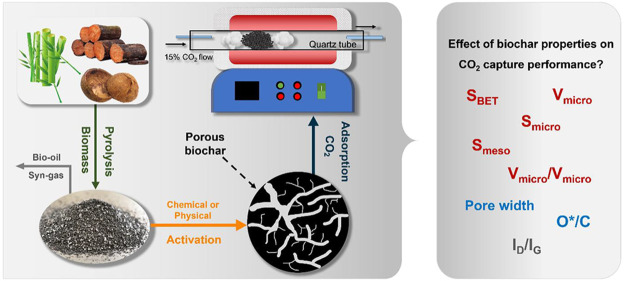

This work investigates three types of biochar (bamboo charcoal, wood pellet, and coconut shell) for postcombustion carbon capture. Each biochar is structurally modified through physical (H_2_O, CO_2_) and chemical (ZnCl_2_, KOH, H_3_PO_4_) activation to improve carbon capture performance. Three methods (CO_2_ adsorption isotherms, CO_2_ fixed-bed adsorption, and thermogravimetric analysis) are used to determine the CO_2_ adsorption capacity. The results show that a more than 2.35 mmol·g^–1^ (1 bar, 298 K) CO_2_ capture capacity was achieved using the activated biochar samples. It is also demonstrated that the CO_2_ capture performance by biochar depends on multiple surface and textural properties. A high surface area and pore volume of biochar resulted in an enhanced CO_2_ capture capacity. Furthermore, the O*/C ratio and pore width show a negative correlation with the CO_2_ capture capacity of biochars.

## 1 Introduction

Climate change is a key challenge nowadays, and CO_2_ emissions are responsible for this challenge.^1^ For example, compared to the preindustrial period, the concentration of CO_2_ in the air has increased by more than 54.4%.^2^ After the United Nations Climate Change Conference 2015, reducing carbon emissions and capturing atmospheric CO_2_ are the main routes to control the global temperature rise.^3^ Therefore, developing technologies to restrain CO_2_ emissions and reduce atmospheric CO_2_ concentrations to achieve carbon neutrality is essential.

Liquid adsorbents, such as liquid amine and aqueous alkali, are widely applied in industries.^4^ However, the volatile loss and corrosion to the instrument are the major problems of liquid adsorbents.^5^ Hence, to explore the further feasibility of CO_2_ adsorbents, solid materials have been used in the carbon capture process.^6^ Solid adsorbents, such as metal–organic framework (MOF),^7,8^ zeolite,^9^ and carbonous materials,^1^ are promising because of their properties of high CO_2_ capacity, easy modification, low cost, and good stability.

Carbonous materials are environmentally friendly sorbents among the reported solid adsorbents. In particular, biochar, as one of the carbonous materials, is generated from biomass pyrolysis under inert conditions.^10^ Biomass from agricultural waste and urban waste as the precursor of biochar has a considerable production globally, promoting biochar production in large quantities.^11^ Additionally, biochar exhibits advantages in environmental protection, application stability, and sustainable development. Therefore, with the superiority of production and adaptability, biochar has been applied in different fields, like catalytic support/catalysis,^12^ water purification,^13^ soil amendment,^14^ and CO_2_ capture.^15^

Compared with other typical solid CO_2_ adsorbents, biochar mainly relies on physical properties to achieve adsorption, transportation, and storage of CO_2_. It is estimated that biochar could capture the amount of greenhouse gas by almost a unit gigaton each year, and nearly 10% of global emissions could be reduced if biomass and biochar are applied for carbon neutrality.^16^ This remarkable environmental benefit is mainly due to 1) large-scale biomass production worldwide, providing adequate sources for biochar materials;^11^ 2) biomass is a carbon-neutral material, having the ability to achieve carbon fixation, and biochar has almost no adverse impact on the environment; and 3) the stability and recyclability of biochar during CO_2_ capture.

Furthermore, the porosity of biochar is one of the requirements for CO_2_ capture, and micropores determining the CO_2_ capture performance of biochar has been widely recognized.^17,18^ Therefore, biochar activation is an essential step in enhancing the porous structure. The main biochar activation methods are physical activation and chemical activation. Physical activation is attained by introducing gases (e.g., CO_2_, H_2_O) to react with biochar under high-temperature conditions and removing the volatile to generate abundant porosity.^19^ Although physical biochar activation is more moderate and less polluting, its activation strength is lower than that of the chemical activation method. In contrast, chemical activation of biochar relies on the reaction between active chemical compounds (e.g., alkaline, acid, molten salts) and carbon to achieve the purpose of porous formation.^20^ However, chemical activation conditions should be carefully controlled to avoid the destruction and structure collapse of biochar due to the high activity of activation agents.^21^ Furthermore, although the physical adsorption of CO_2_ by biochar is the main pathway, surface modification of biochar is popular in enhancing CO_2_ chemical adsorption. Either amine grafting or nitrogen dope makes up for the lack of nitrogen in biochar materials.

Nevertheless, there are still challenges for biochar-based carbon capture. Notably, the process is mainly dependent on the porous structure of biochar. There are unknowns about the influence of textural properties of biochar on CO_2_ capture. By contrast, biochar shows less uniformity than hydrothermal char because of the complex components of feedstocks and precursors.^22,23^ In addition, in the choice of biochar activation method, much of the work states that chemical activation is better but lacks a comparison with physical activation.^24,25^ Correlation analysis of biochar properties and the relation to the CO_2_ capture performance could be essential to help promote technology development.

Herein, this work investigated the influence of biochar activation on the CO_2_ capture performance obtained by TGA, the KANE 457 portable gas analyzer, and the ASAP 2020 gas analyzer. Moreover, three lignocellulosic biochar samples were used to explore the CO_2_ adsorption performance of different biochar feedstock. The activation agents, including ZnCl_2_, H_3_PO_4_, and KOH, were also investigated. Therefore, this work explores a potential mechanistic explanation of the CO_2_ adsorption process regarding the influence of pore size, surface properties, and biochar preparation and activation.

## 2 Materials and Methods

### 2.1 Materials and Biochar Activation

**Figure 1:**
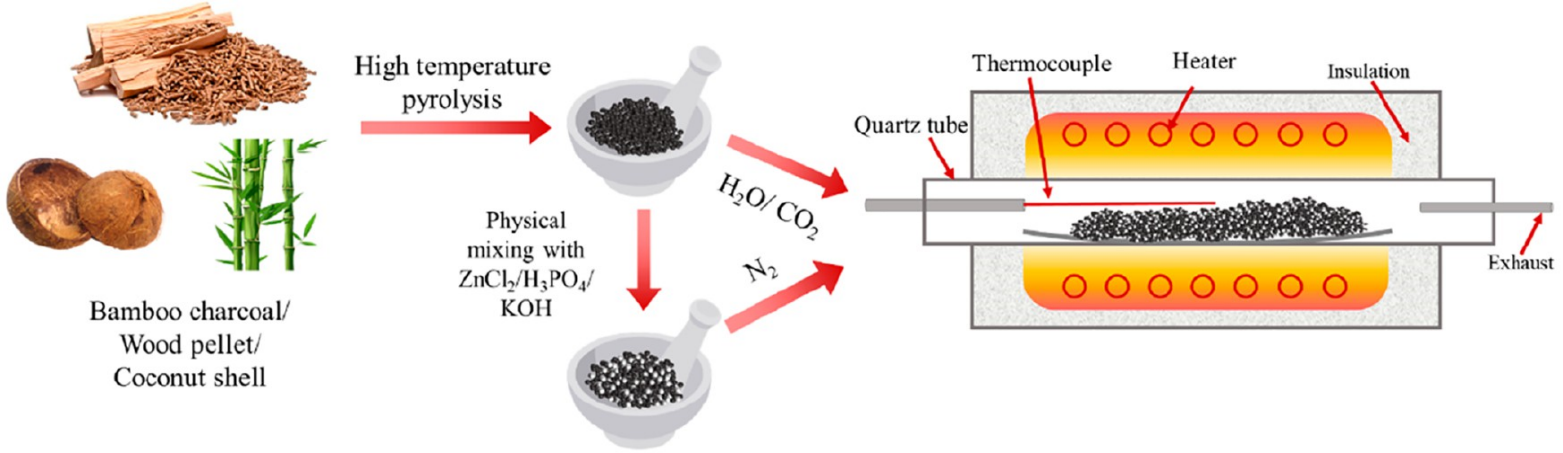
Schematic diagram for biomass pyrolysis and biochar activation.

KOH (Sigma-Aldrich, ≥85%), ZnCl_2_ (Sigma-Aldrich, ≥95%), and H_3_PO_4_ (Sigma-Aldrich, 58 wt % in H_2_O) were utilized as the activator for biochar activation processes. The lignocellulosic biochar samples used in this work included bamboo charcoal biochar (BC-Origin), wood pellet biochar (WP-Origin), and coconut shell biochar (CS-Origin). Bamboo charcoal (BC) was obtained from the pyrolysis of compressed bamboo sawdust with a high density at ∼1000 °C from Ken Chiku Company. Wood pellet (WP) was produced from EN1 grade A wood pellets from Enertecgreen Ltd. And coconut shell (CS) was carbonized from coconut shell fragments at high temperatures (∼1200 °C) in an inert atmosphere by Lvzhiyuan Company. Figure 1 shows the pyrolysis and biochar activation processes, and the detailed steps of specific activation methods are listed separately below.

Steam activation: as the previous work reported,^18^ the biochar was sieved to less than 500 μm and dried overnight at 110 °C in an oven. A sample weighing approximately 6 g was placed in a horizontal quartz tube and heated at a rate of 10 °C min^–1^ under a nitrogen flow (100 mL min^–1^). Once the temperature reached 250 °C, 15 mL of deionized water was continuously injected at a rate of 10 mL h^–1^ to introduce steam into the quartz tube. Set the activation temperature to 550 °C and the duration to an hour. After the activated sample cooled to room temperature, it was washed with deionized water and dried in an oven overnight, and the samples were labeled as BC-H_2_O, WP-H_2_O, and CS-H_2_O.

CO_2_ activation: approximately 6 g of sieved and dried biochar was pretreated in quartz under nitrogen flow rates of 100 mL min^–1^. When the temperature reached 250 °C, the gas path was switched to CO_2_. The CO_2_ activation was sustained for one h at 550 °C. After the system cooled down to room temperature, the samples were washed with deionized water, dried in the oven overnight, and named BC-CO_2_, WP-CO_2_, and CS-CO_2_, respectively.

Chemical activation: According to the previous works,^26,27^ equal mass amounts of dried biochar and chemical compounds (ZnCl_2_, H_3_PO_4_, or KOH) were agitated for 24 h at room temperature in 40 mL of deionized water. The chemically impregnated biochar samples were sieved and dried overnight at 110 °C. The dried chemically impregnated sample was placed into a quartz tube and exposed to 100 mL·min^–1^ nitrogen. The furnace was heated at 10 °C·min^–1^ until it reached 550 °C, which remained for 1 h. After the biochar was allowed to cool to room temperature, 0.1 mol·L^–1^ HCl (NaOH for removing H_3_PO_4_) and sufficient deionized water were used to dislodge water-soluble ions. The activated biochar samples were dried overnight at 110 °C and then sieved under a mesh size of 500 μm. The prepared samples are labeled as BC-ZnCl_2_, BC-KOH, BC-H_3_PO_4_, WP-ZnCl_2_, WP-KOH, WP-H_3_PO_4_, CS-ZnCl_2_, CS-KOH, and CS-H_3_PO_4_, respectively.

### 2.2 Biochar Characterization

The content of C, H, N, and S in the biochar samples was determined by a PerkinElmer PE2400 CHSN Element Analyzer. The proximate analysis (measuring the content of ash, moisture, fixed carbon, and volatile matter of biochar) was tested by the TGA 2950 Thermogravimetric Analyzer. The compositions of Ca, K, P, and Si were measured by inductively coupled plasma-optical emission spectroscopy (ICP-OES). X-ray diffraction (XRD) patterns were obtained with a PANalytical Empyrean Series 2 diffractometer with a Cu Ka X-ray source. The presence of surface functional groups and aromatic groups of biochar in the range of 3000–1400 cm^–1^ was determined by an Agilent Cary 630 FTIR spectrometer. Scanning electron microscopy (SEM) was performed with a Quanta FEG 250 at 20 keV voltage under a high chamber vacuum. Raman spectroscopy was obtained with a WItec Alpha 300 Raman microscope. The microscope was equipped with a 100× lens and a 532 nm laser. The Micromeritics ASAP 2020 characterized the textural properties of biochar samples at 77 K. The surface area was obtained using the Brunauer–Emmett–Teller (BET) method. The porosity of samples was obtained using the t-Plot method, Barrett–Joyner–Halenda (BJH) method, and Horvath–Kawazoe method. CO_2_ and N_2_ adsorption isotherms were also investigated by the Micromeritics ASAP 2020 gas adsorption analyzer at 298 K after degassing at 200 °C for 6 h.

### 2.3 CO_2_ Capture Capacity

Kane 457 Gas Analyzer: ∼1 g of a biochar sample was placed in the quartz tube (OD: 12 mm) sandwiched by quartz wool, and 15% CO_2_ balanced in N_2_ was introduced into the system at a flow rate of 100 mL·min^–1^. The adsorption and desorption of CO_2_ using the biochar samples were achieved by the temperature swing method, which measured the CO_2_ capture at room temperature and CO_2_ release at 70 °C. Based on the variation of CO_2_ concentration at the end of the fixed bed, the CO_2_ capture capacities of biochar can be calculated according to the following equation
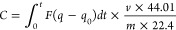
1where *C* means the CO_2_ capture capacity of biochars; *q* and *q*_0_ are outlet and inlet CO_2_ concentrations; *t* is the equilibrium time; *v* is the 15% CO_2_ flue rate; and *m* is the actual biochar mass placed in the quartz tube.

TGA 2950 Thermogravimetric Analyzer: around 20 mg of a specified biochar sample was placed into the 50 μL platinum sample pan, and the balance purge was continuously fed with nitrogen at 100 mL min^–1^. The sample was preheated at 110 °C for 20 min under nitrogen, and the furnace purge was switched to 15% CO_2_ at the flow of 100 mL·min^–1^ when the furnace temperature decreased to 25 °C. The instrument was warmed to 70 °C to remove the captured CO_2_ after the mass signal stabilized.

## 3 Results and Discussion

### 3.1 Biochar Characterization

#### 3.1.1 Proximate Analysis

**Figure 2:**
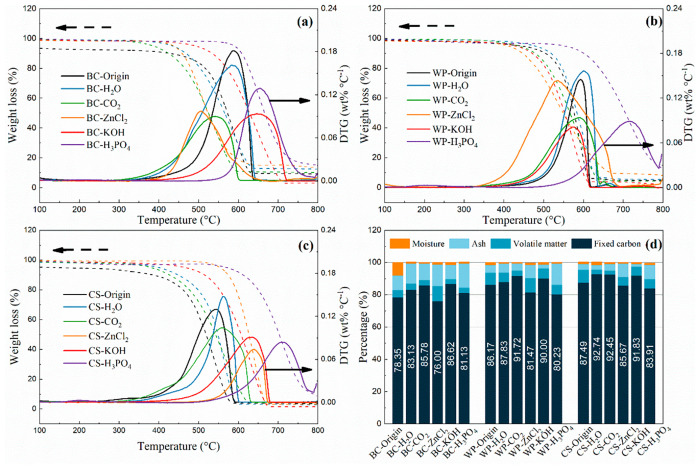
TGA-TPO and TGA-DTG profiles of (a) bamboo charcoal, (b) wood pellet, and (c) coconut shell; (d) biochar composition determined by proximate analysis.

Figure 2 shows the results of the temperature-programmed oxidation (TPO) of biochars and proximate analysis. TPO allows the evaluation of the thermochemical conversion of biochar, thus providing information on the oxidative stability of carbonaceous solids, where the reactivity at higher temperatures indicates a more stable and ordered structure.^28^ Typically, the oxidation temperatures of three initial biochars are in the range 400–650 °C. The oxidation temperature of WP is around 550 °C, which is relatively higher than that of other feedstock. For the CS biochars after the activation, the DTG peaks (Figure 2(c)) are all shifted to higher temperatures, indicating that the activation enhances the thermal stability of the CS biochars, which might be related to the small particles of CS that could sufficiently react with the activators. Furthermore, in terms of the effect of activators on the TPO of the samples, chemical activation seems to increase the oxidation temperature range of the biochar samples. Moreover, the H_3_PO_4_ activation expands the oxidation peak to nearly 700 °C, illustrating a higher biochar thermal stability.^29^ Notably, although the production of biochar from biomass has eliminated a large number of volatile substances through high-temperature pyrolysis, phosphoric acid as an activator can still interact with the residual organic matter to form phosphate and polyphosphate groups, prompting the activation process to swell and expand the porous structure.^30^

Proximate analysis can also assess biochar stability and composition.^31^Figure 2(d) shows the proximate analysis results of the biochar samples. Fixed carbon is one of the indicators to determine the advantages of biochar, and most of the biochar has a higher fixed carbon content after activation, except for six samples activated with H_3_PO_4_ and ZnCl_2_. For the molten salt activation, a higher volatile fraction of biochar after ZnCl_2_ activation was confirmed.^32^ This might be due to the aromatization between ZnCl_2_ and the residual cellulose-like structure inside the biochar during the activation process, resulting in structural changes.^33^ In addition, the proportion of fixed carbon after phosphoric acid activation is reduced. It is suggested that the activation of biochar under acidic conditions generates acidic functional groups, increasing oxygen content and reducing the proportion of fixed carbon.^34^

#### 3.1.2 CHNS Results

The elemental analysis results for determining C, H, N, and S percentages (%) are summarized in Table 1. According to Table 1, biochar samples have considerable amounts of carbonous and oxygenated compounds. The low concentration of nitrogen element proves that the activated biochar lacks nitrogen-containing functional groups, which may result in insufficient chemisorption of carbon dioxide by biochar materials.^35^ In addition, compared to the proximate analysis (Figure 2(d)), the elemental analysis obtains similar results: 1) the activated biochars have a higher carbon content than the precursors, except for the phosphoric acid and molten salt activated biochars; 2) the higher oxygen content after H_3_PO_4_ activation indicates that more oxygen-containing functional groups are generated and attached to the surface of the biochar; and 3) as the oxygen content is obtained by difference, it may include unremoved metals, which is consistent with the increased ash content of the ZnCl_2_ activated biochars.

**Table 1:** CHNS Analysis Results

**Name**	**C**	**H**	**N**	**S**	O*^a^
**BC-Origin**	78.05	2.10	0.34	1.17	18.34
**BC-H**_**2**_**O**	82.51	0.81	<0.30	<0.30	16.08
**BC–CO**_**2**_	84.18	1.97	<0.30	<0.30	13.25
**BC-ZnCl**_**2**_	74.26	1.32	<0.30	<0.30	23.82
**BC-KOH**	84.46	2.25	<0.30	<0.30	12.69
**BC-H**_**3**_**PO**_**4**_	78.97	1.24	0.45	<0.30	20.04
**WP-Origin**	88.30	1.35	<0.30	0.43	9.67
**WP-H**_**2**_**O**	90.50	1.06	<0.30	<0.30	7.84
**WP-CO**_**2**_	91.15	0.68	<0.30	<0.30	7.57
**WP-ZnCl**_**2**_	76.42	0.91	<0.30	<0.30	22.07
**WP-KOH**	88.51	1.18	<0.30	<0.30	9.71
**WP-H**_**3**_**PO**_**4**_	75.12	0.87	<0.30	<0.30	23.41
**CS-Origin**	83.88	1.22	<0.30	<0.30	14.3
**CS-H**_**2**_**O**	90.65	1.32	<0.30	<0.30	7.43
**CS-CO**_**2**_	89.77	0.87	<0.30	<0.30	8.76
**CS-ZnCl**_**2**_	90.93	0.58	<0.30	<0.30	7.89
**CS-KOH**	90.47	0.88	<0.30	<0.30	8.05
**CS-H**_**3**_**PO**_**4**_	75.86	1.13	<0.30	<0.30	22.41

a O* is obtained by difference.

#### 3.1.3 FTIR

**Figure 3:**
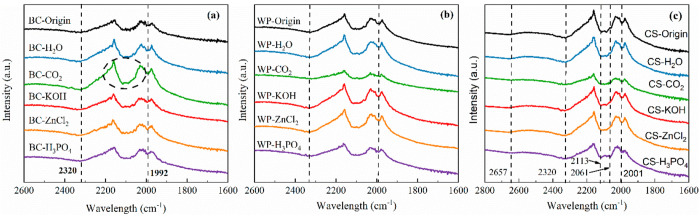
FTIR spectra of (a) bamboo charcoal biochars, (b) wood pellet biochars, and (c) coconut shell biochars.

The ATR-FTIR spectra in the wavelength range 2600–1600 cm^–1^ are shown in Figure 3. Overall, the FTIR results are similar among the different samples. A strong appearance at 2320 cm^–1^ indicates the O=C=O stretching of CO_2_, and the weak aromatic C–H bending is indicated at 2000–1990 cm^–1^.^36^ Moreover, the peaks from 2200 to 2000 cm^–1^ (circled in Figure 3(a)) correspond to the central double bond groups (e.g., C=N, C=C=C).^18^ Briefly, for the same biochar species, the profiles of the samples do not change significantly after activation under different conditions, which indicates that the activated biochar was only structurally altered.

#### 3.1.4 SEM

**Figure 4:**
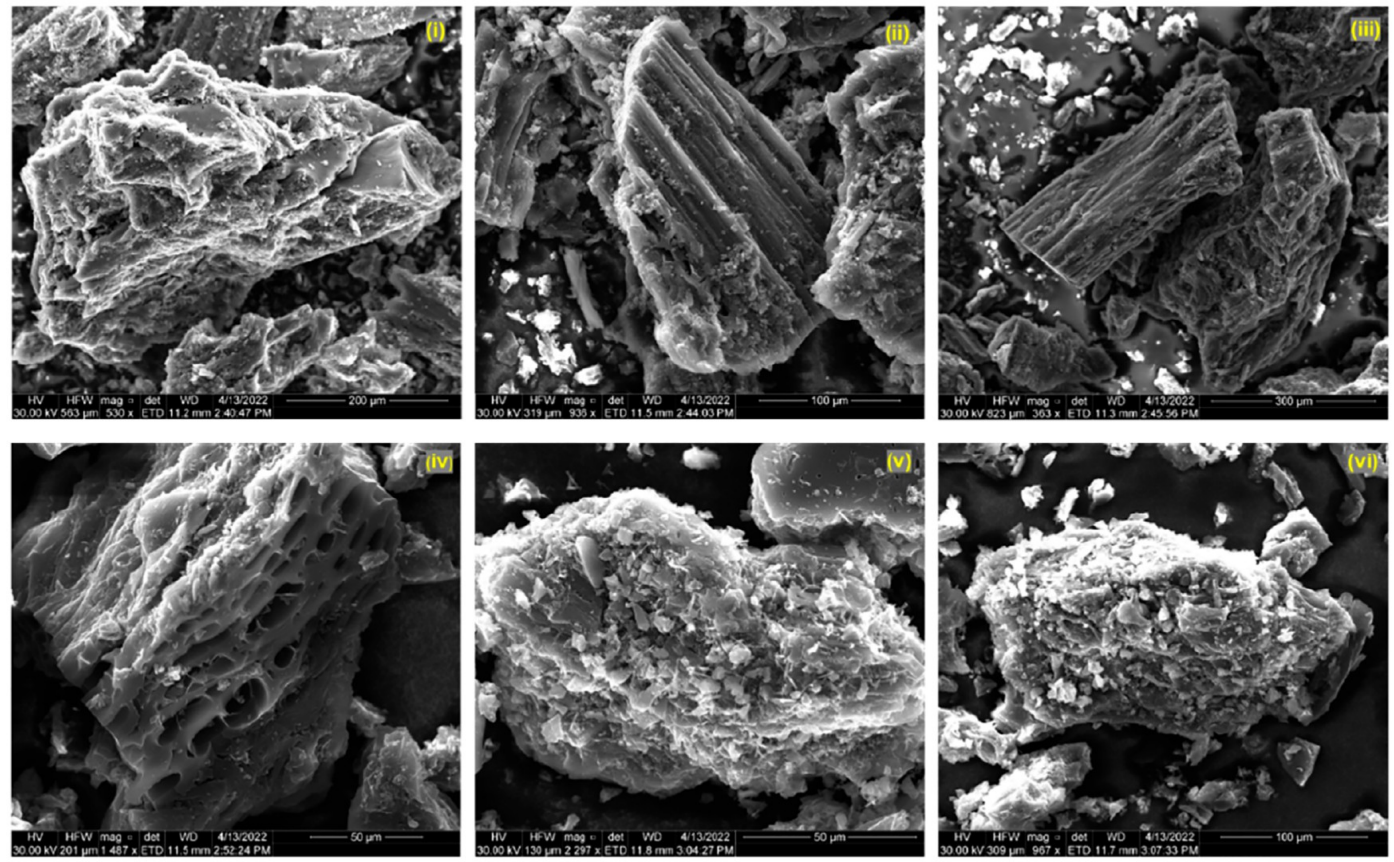
SEM images of bamboo charcoal biochars, sample logs: original biochar (i) and activated by H_2_O (ii), CO_2_ (iii), ZnCl_2_ (iv), KOH (v), and H_3_PO_4_ (vi).

**Figure 5:**
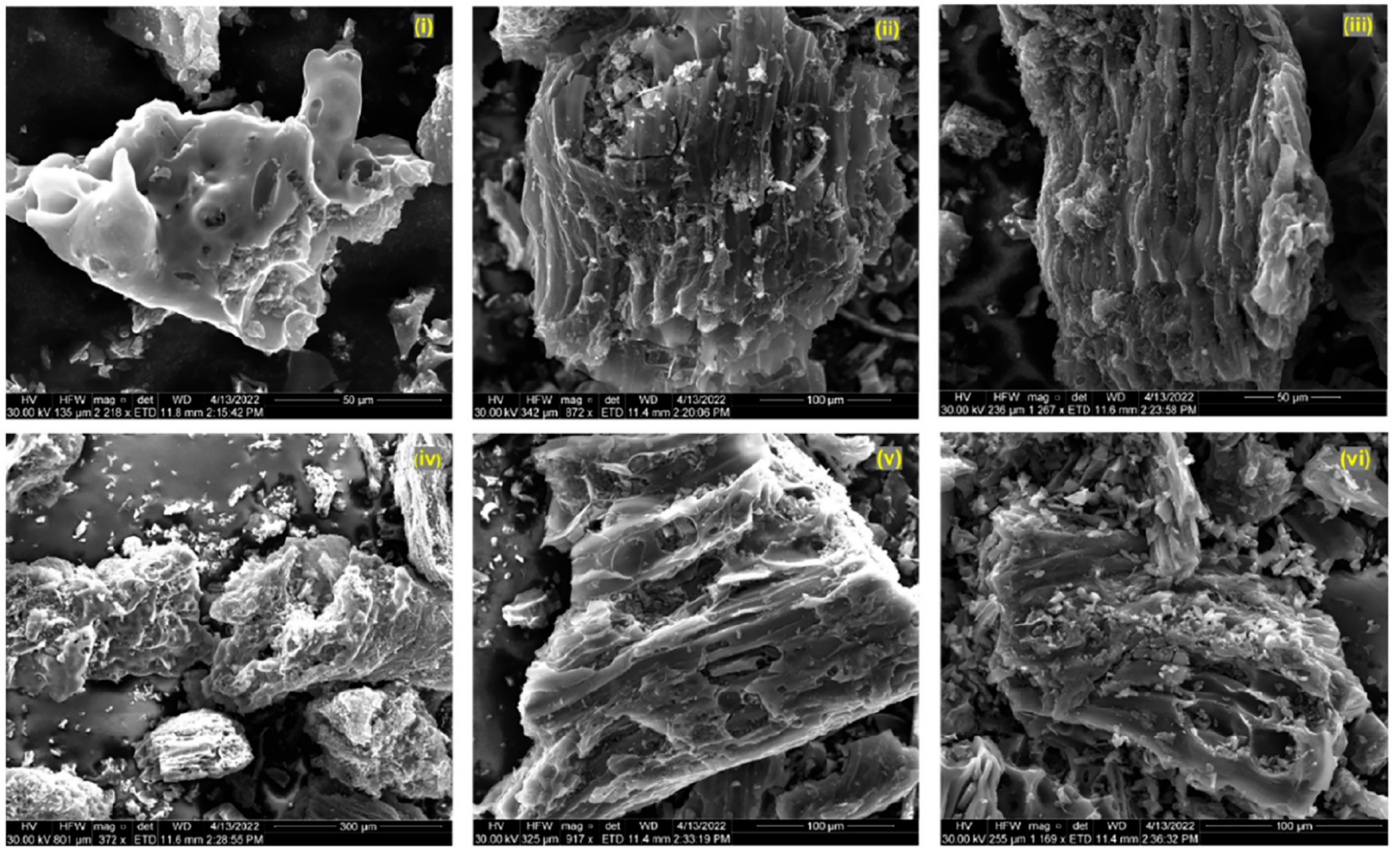
SEM images of wood pellet biochars, sample logs: original biochar (i) and activated by H_2_O (ii), CO_2_ (iii), ZnCl_2_ (iv), KOH (v), and H_3_PO_4_ (vi).

**Figure 6:**
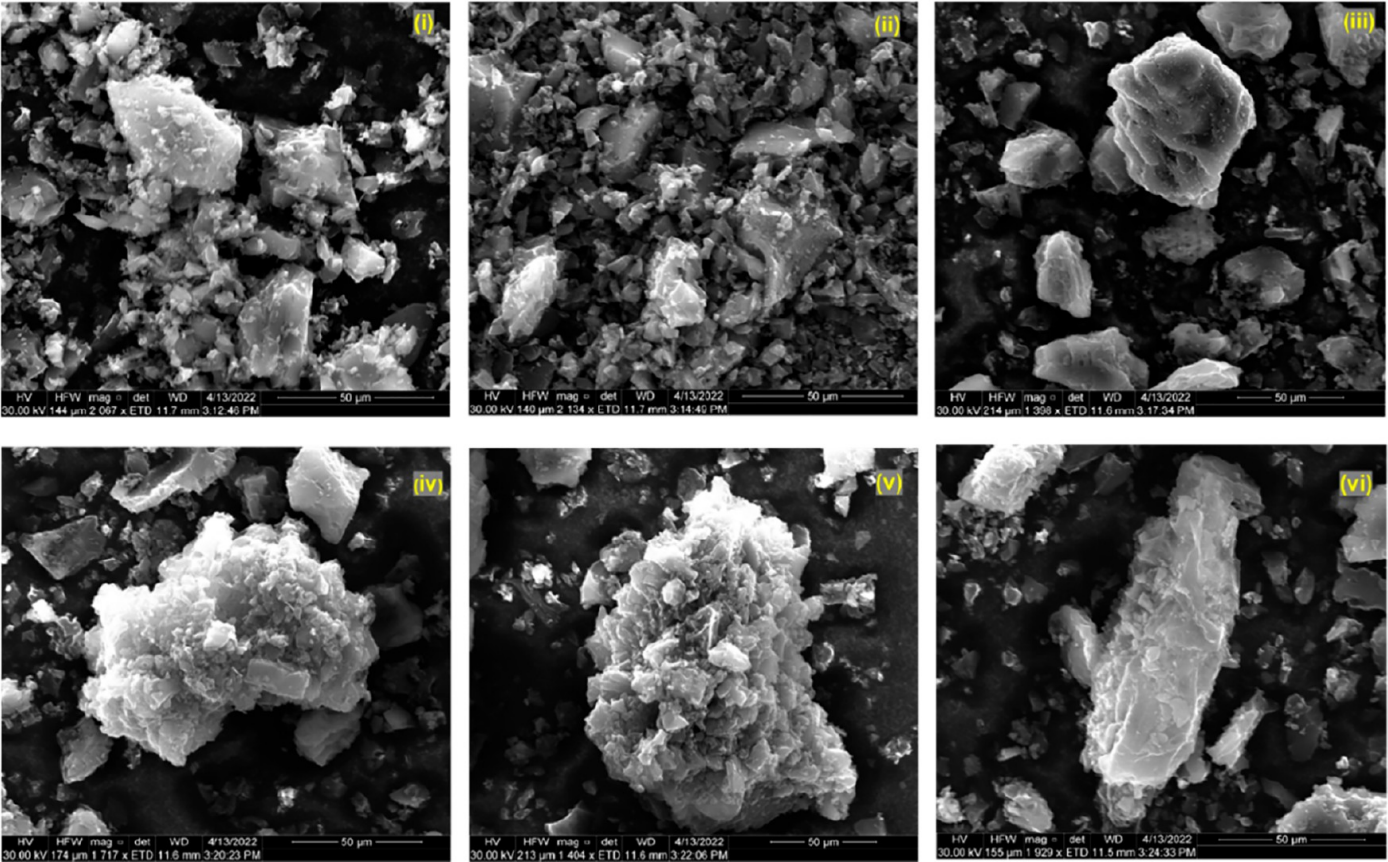
SEM images of coconut shell biochars, sample logs: original biochar (i) and activated by H_2_O (ii), CO_2_ (iii), ZnCl_2_ (iv), KOH (v), and H_3_PO_4_ (vi).

Figures 4–6 demonstrate the morphologies of the biochars analyzed by scanning electron microscopy (SEM). Essentially, the BC and WP biochars have similar structures, and their particles are relatively large compared to the CS biochar, which is due to the more stringent pyrolysis conditions of CS. For the CS biochar, although the small particle size of the biochar facilitates an adequate reaction with the activators, it is challenging to enlarge the pore size presented in the precursor due to its inherent fragmentation. Moreover, it is also difficult to observe the micropore structure on SEM scales. On the contrary, the BC and WP biochars are easier to characterize. The physical activation of biochar (steam activation and CO_2_ activation) mainly results in the tubular pore structure,^37^ while chemical activation produces pore expansion and corrosion.^38^ From Figures 4(iv)–(vi) and 5(iv)–(vi), the irregular collapse of the pores shows that the chemical activation made the surface of the biochar rougher. Additionally, despite attempts to wash the biochars with acid and alkaline solutions after activation, it is evident that the surface of the biochar activated using metal hydroxide and molten salt has a metallic crystalline structure (Figures 6(iv) and 6(v)). In particular, the CS biochars are completely covered with metal-containing compounds on the surface. This phenomenon is also detected by XRD (Figure S1). In addition, the XRD results show metallic signals in addition to the conventional graphite peak signal.^39^

#### 3.1.5 Raman Spectroscopy

**Figure 7:**
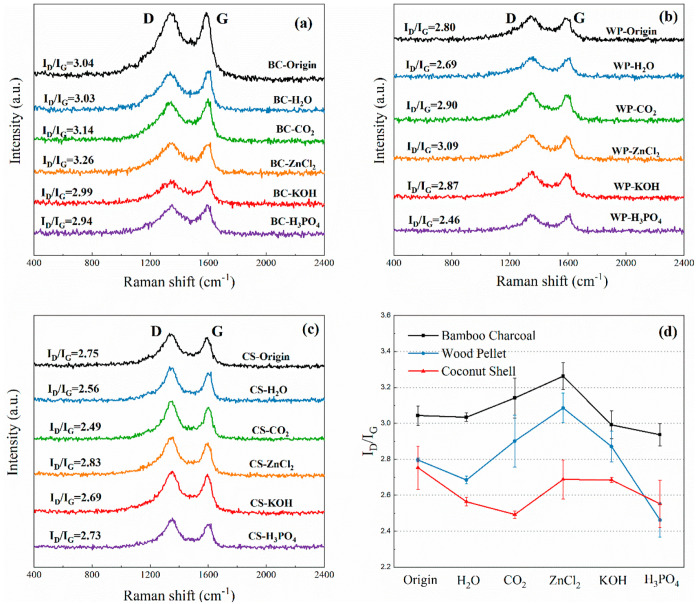
Raman spectra of (a) bamboo charcoal; (b) wood pellet; and (c) coconut shell biochar fragments. All of the spectra show the D and G bands, indicating graphitic structures and defected graphitic structures, respectively; (d) I_D_/I_G_ band ratio trend of bamboo charcoal, wood pellet, and coconut shell biochar.

Raman spectra of the biochar samples are shown in Figure 7, which confirms the presence of G-band (1580–1600 cm^–1^ from vibrations of graphitic sp^2^ carbon) and D-band (1350–1370 cm^–1^ from vibrations of sp^2^ bonded defected carbon).^40^ The ratio of I_D_ and I_G_ can be regarded as the order of magnitude of carbon rings for biochar adsorbents. The biochar samples in this work show fewer disordered carbons (I_D_/I_G_ > 2.00), confirming the presence of amorphous carbon as demonstrated by the XRD results (Figure S1). In addition, Figure 7(d) shows that different samples have a similar overall trend in I_D_/I_G_, while the activation seems to affect the orderliness of the biochar carbon. For example, ZnCl_2_ activation increases the disorder of carbon in the BC and WP biochars, while that in H_3_PO_4_ is the opposite.

#### 3.1.6 Porous Analysis

Table 2 presents the results of the porous analysis, including surface area, pore volume, and pore width. It is essential to activate biochar to enhance the surface properties for CO_2_ capture. However, ZnCl_2_ and H_3_PO_4_ activation methods have a negative effect on improving the porosity of biochar. The most effective activation method in terms of the specific surface area is KOH activation, which expands the specific surface area of the BC biochar from 91.17 m^2^·g^–1^ to 526.36 m^2^·g^–1^. Similarly, the other two biochars activated by KOH under the same activation conditions demonstrated larger specific surface areas. Likewise, the microporous structure of the activated biochar is enhanced after the activation, which can be supported by the ratio of S_micro_/S_meso_ and V_micro_/V_total_. Moreover, it is worth mentioning that KOH activation is more favorable to generating micropores because of the reaction between KOH and C during the activation process and the introduction of a metallic frame into the carbon matrix.^41^

**Table 2:** BET Surface Area, Micropore/Mesopore Surface Area, Micropore/Total Volume, and Average Pore Diameter of the Prepared Sorbents

Name	S_BET_^a^ (m^2^ g^–1^)	S_micro_^b^ (m^2^ g^–1^)	V_micro_^b^ (cm^3^ g^–1^)	S_meso_^b^ (m^2^ g^–1^)	V_total_^c^ (cm^3^ g^–1^)	Pore width^c^ (nm)
BC-Origin	91.17	47.46	0.03	32.74	0.17	3.40
BC-H_2_O	191.22	89.34	0.05	80.28	0.29	6.05
BC–CO_2_	174.09	60.75	0.01	114.49	0.29	6.65
BC-ZnCl_2_	96.93	10.25	0.01	78.09	0.17	7.10
BC-KOH	526.36	343.14	0.16	131.29	0.46	3.47
BC-H_3_PO_4_	85.52	4.80	0.01	67.15	0.16	7.64
WP-Origin	160.66	145.18	0.08	15.48	0.10	4.36
WP-H_2_O	306.99	276.89	0.13	30.10	0.16	3.49
WP-CO_2_	287.02	262.46	0.13	24.56	0.15	2.73
WP-ZnCl_2_	4.56	4.10	0.002	0.46	0.01	16.03
WP-KOH	438.72	387.45	0.20	51.27	0.24	3.54
WP-H_3_PO_4_	3.19	2.29	0.001	0.90	0.01	5.45
CS-Origin	1252.62	949.53	0.42	303.09	0.61	2.74
CS-H_2_O	1315.61	980.80	0.44	326.66	0.64	2.70
CS-CO_2_	1270.45	923.41	0.42	347.04	0.64	2.76
CS-ZnCl_2_	1297.51	908.61	0.42	388.89	0.65	2.73
CS-KOH	1352.60	1017.23	0.45	335.37	0.67	2.86
CS-H_3_PO_4_	983.39	762.35	0.34	221.03	0.53	3.21

a BET method.

b t-plot method.

c Single point method.

**Figure 8:**
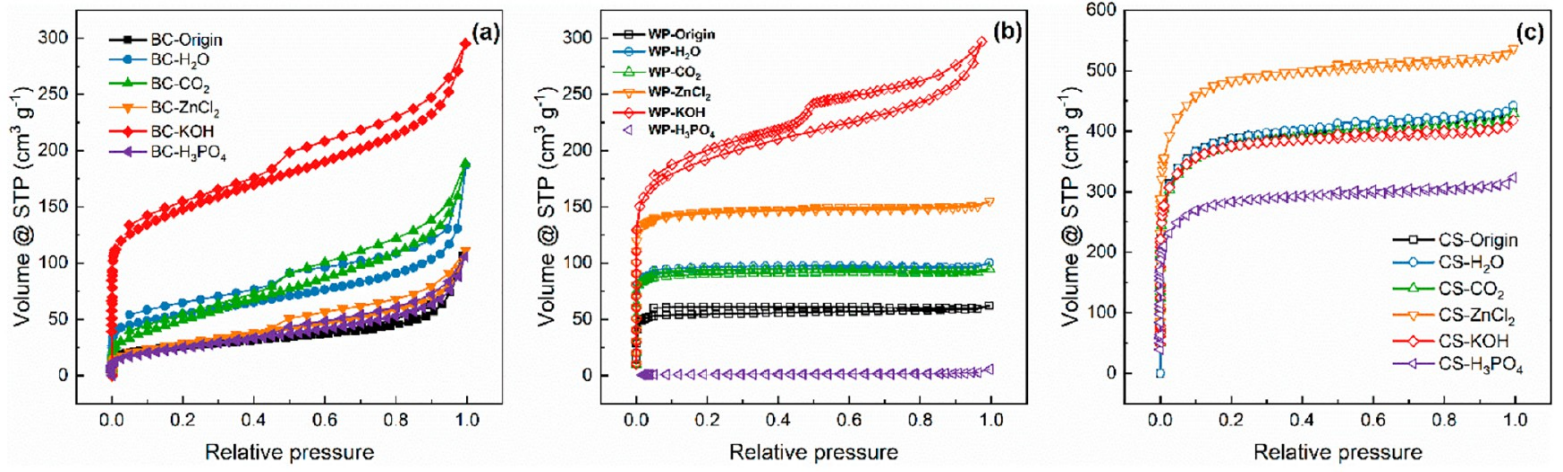
N_2_ adsorption–desorption isotherms of (a) bamboo charcoal biochars, (b) wood pellet biochars, and (c) coconut shell biochars.

Furthermore, CS biochar has the most complex surface structure (1252.62 m^2^·g^–1^) and a large number of microporous structures. However, if a high temperature was used to carbonize biomass, a smaller particle size of biochar could be generated. The nitrogen adsorption–desorption linear isotherm plots (Figure 8) show that the CS biochar demonstrates a type I BET isotherm curve, which indicates a predominantly microporous structure.^42^ The BC biochar that fits the type IV isothermal curve demonstrates a mesoporous structure,^43^ while the WP biochar has a relatively insignificant porous structure. Moreover, the surface areas of WP and BC are decreased after H_3_PO4 and ZnCl_2_ activation, which might be caused by the residual activators and P-containing functional groups or the collapse and shrinkage of pores for these activation methods under the experimental temperature.^24^

**Figure 9:**
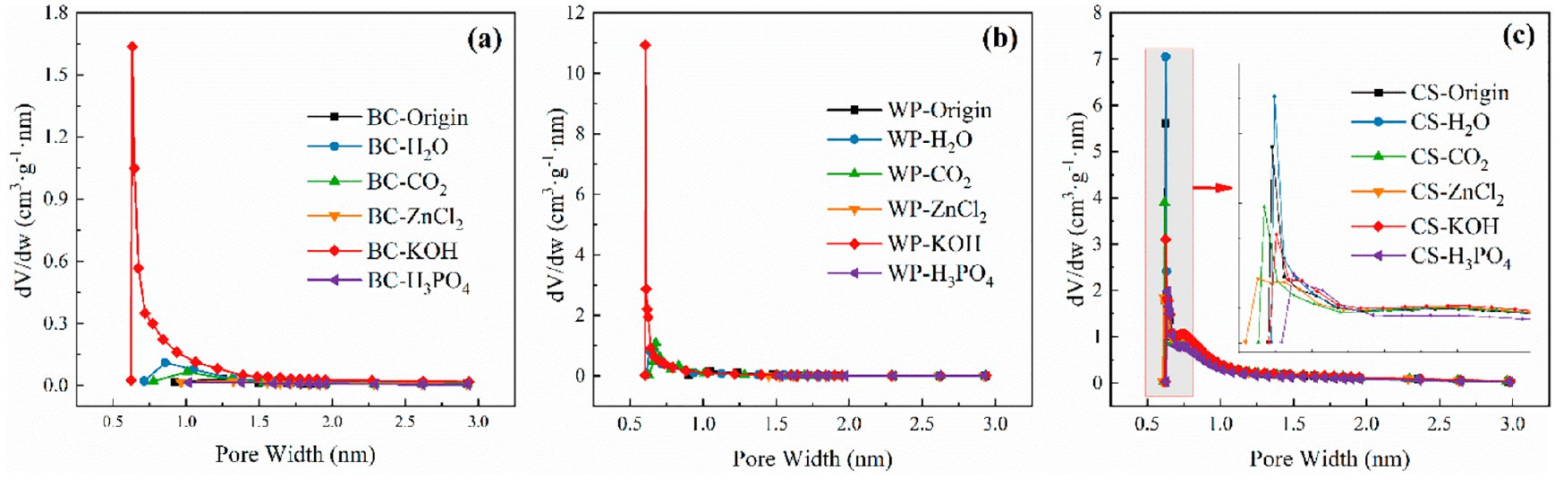
Horvath–Kawazoe differential pore volume plot of (a) bamboo biochars, (b) wood pellet biochars, and (c) coconut shell biochars.

It is known that biochar relies on the physical adsorption of micropores for the CO_2_ capture. Therefore, the pore size distribution of the micropores is derived in Figure 9 according to the Horvath–Kawazoe method. According to Figure 9(c) and Table 2, the CS biochar mainly has micropores. Microporous structures are dominant only in the BC and WP biochar when KOH is used for activation.

### 3.2 CO_2_ Capture Capacity

**Figure 10:**
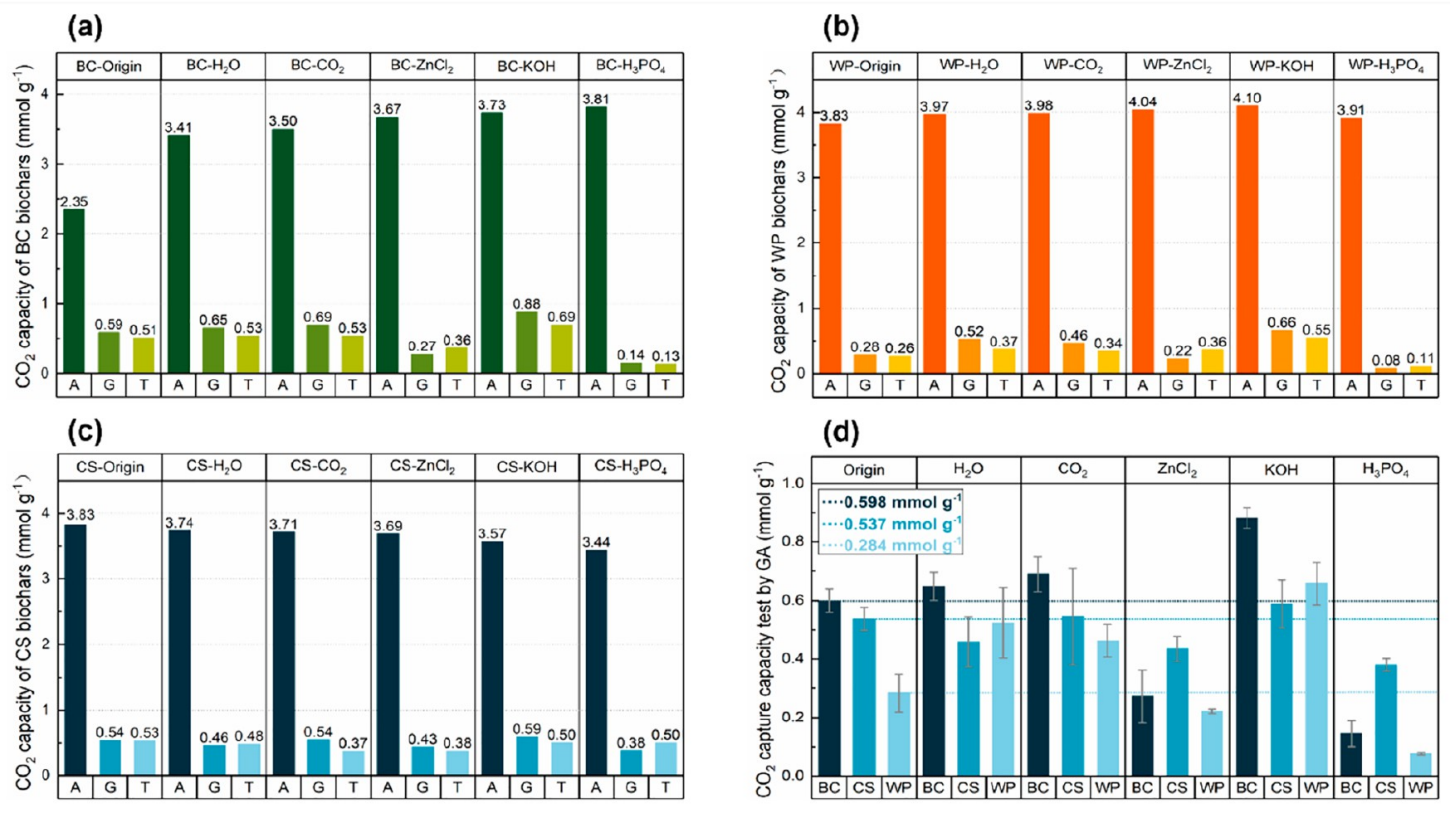
CO_2_ capture capacities tested by the ASAP 2020 (A), Kane 457 gas analyzer (G), and thermal gravity instrument (T) of (a) bamboo charcoal biochars, (b) wood pellet biochars, and (c) coconut shell biochars; (d) Comparison of different activation methods on CO_2_ capacity performance of biochar samples.

The CO_2_ capture by biochar was measured with three instruments: the ASAP 2020 (A), KANE 457 gas analyzer (G), and thermogravimetric analysis (T). The three instruments have different advantages and disadvantages. For example, the ASAP 2020 can determine the adsorption capacity of biochar at a specific partial pressure. Figure 10 represents the adsorption capacity of pure carbon dioxide at 1 bar and 298 K, which is why the values of A are higher than those of G and T. The thermogravimetric test used 15% CO_2_ as the carbon source (balanced with N_2_); this process might be affected by the adsorption of N_2_. The KANE 457 Gas Analyzer monitors the change in the CO_2_ concentration in the system via an infrared detector, allowing a more accurate calculation of the CO_2_ capture capacity by biochars.

As shown in Figure 10, the different biochar capture capacities measured in the three ways have a generally consistent trend, in terms of CO_2_ adsorption. Therefore, Figure 10(d) presents the effect of the different activation methods on the adsorption of CO_2_ from the KANE 457. It is demonstrated that the H_2_O, CO_2_, and KOH activation methods enhance the ability of CO_2_ adsorption using the WP and BC biochars. For example, the CO_2_ adsorption capacity of KOH-activated BC increases from 0.59 mmol g^–1^ to 0.88 mmol g^–1^, and the adsorption capacity of WP biochar also increases by 85.7% after water vapor activation. However, not all activations enhance the performance of biochar, in terms of CO_2_ adsorption. ZnCl_2_ and H_3_PO_4_ activations substantially reduce the carbon capture performance of the three biochars. This may be because of (i) the collapse of pores during the activation and (ii) the increase of surface acidity of the biochar (introducing P-containing functional groups),^24^ making it challenging to capture the acidic gas of CO_2_. Therefore, although activation can improve the pore structure of biochar, increasing the CO_2_ capture capacity of biochar is also subject to other factors of CO_2_ capture, such as the surface functionalities of biochar.

### 3.3 CO_2_/N_2_ Selectivity

The selectivity of biochar for CO_2_ and N_2_ is one of the critical criteria for carbon dioxide capture by biochar. The ideal adsorbed solution theory (IAST) theory^44,45^ is based on Langmuir fit CO_2_ and N_2_ isotherm adsorption at the same conditions. The CO_2_/N_2_ selectivity can be calculated according to the parameter data of eq 2, where *q_m_* (mmol g^–1^) is the theoretical saturated adsorption ability, and *K_L_* is the constant for the Langmuir equation. Figure S4 shows the isothermal sorption of carbon dioxide and nitrogen by the biochars at 298 K, and the fitted data for each type of biochar is shown in Table 3. Typically, α (CO_2_/N_2_) greater than 2.0^46^ is considered to indicate that biochar tends to retain CO_2_ and reduce N_2_ adsorption during the adsorption process. CS-H_3_PO_4_ performed with a maximum selectivity of 12.484.
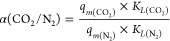
2

**Table 3:** Parameters of the Langmuir Model Fittings for Isotherms of Biochar Samples

	CO_2_				N_2_			
	Capacity at 25 °C, 1 bar (mmol g^–1^)	*q_m_* (mmol g^–1^)	*K_L_* (*101 MPa^–1^)	R^2^	*q_m_* (mmol g^–1^)	*K_L_* (*101 MPa^–1^)	R^2^	α(CO_2_/N_2_)
BC-Origin	3.345	6.521	0.988	0.997	1.564	0.410	0.999	10.047
BC-H_2_O	3.410	6.775	0.990	0.998	2.207	0.294	0.999	10.337
BC–CO_2_	3.501	8.039	0.760	0.999	1.060	0.990	0.997	5.822
BC-ZnCl_2_	3.666	13.738	0.362	0.999	0.977	1.270	0.997	4.008
BC-KOH	3.730	12.612	0.411	0.999	2.254	0.362	0.999	6.353
BC-H_3_PO_4_	3.813	7.433	0.988	0.997	1.439	0.760	0.999	6.715
WP-Origin	3.826	6.610	1.272	0.997	1.234	0.989	0.998	6.889
WP-H_2_O	3.967	7.882	0.991	0.998	2.147	0.411	0.999	8.852
WP-CO_2_	3.984	9.146	0.760	0.998	2.950	0.295	0.999	7.987
WP-ZnCl_2_	4.042	6.984	1.272	0.997	1.061	0.992	0.997	8.440
WP-KOH	4.100	7.992	0.991	0.997	0.994	1.273	0.997	6.259
WP-H_3_PO_4_	3.913	17.179	0.294	0.999	1.234	0.991	0.998	4.130
CS-Origin	3.827	12.939	0.411	0.999	1.624	0.761	0.999	4.303
CS-H_2_O	3.738	7.427	0.991	0.998	1.980	0.362	0.999	10.269
CS-CO_2_	3.714	8.528	0.760	0.999	0.981	0.992	0.997	6.660
CS-ZnCl_2_	3.686	6.367	1.272	0.997	1.584	0.411	0.999	12.440
CS-KOH	3.567	7.086	0.990	0.998	0.906	0.992	0.997	7.805
CS-H_3_PO_4_	3.436	7.888	0.760	0.999	1.960	0.245	0.999	12.484

### 3.4 Correlation Analysis

**Figure 11:**
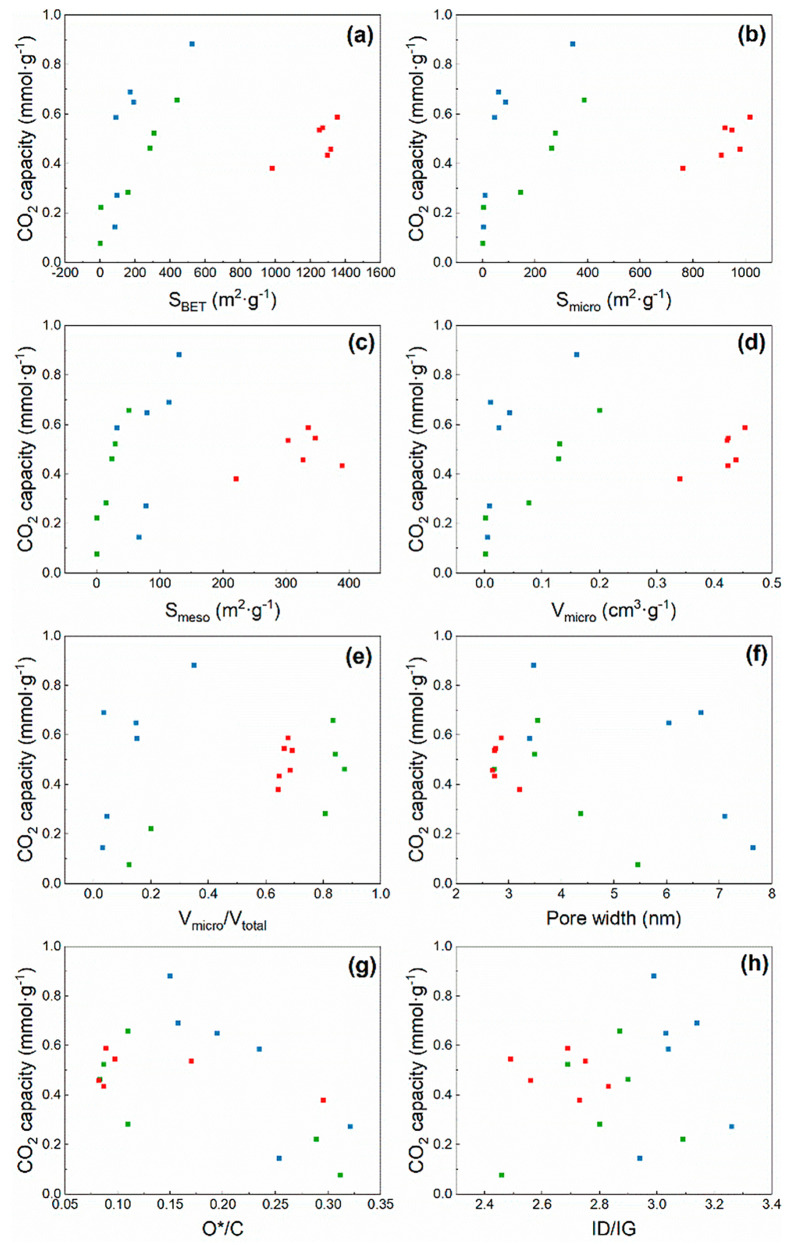
Correlation analysis between the CO_2_ capture capacity obtained by the gas analyzer (BC-blue, WP-green, CS-red) and (a) BET surface area, (b) micropore surface area, (c) mesopore surface area, (d) micropore volume, (e) micropore volume/total pore volume, (f) pore width, (g) *O/C, and (h) I_D_/I_G_.

Correlation analysis between the carbon dioxide capture capacity and various properties provides a more direct indication of whether the properties of the biochar are conducive to implementing carbon capture. Figure 11 shows the relationship between the three biochars and the eight representative properties of biochars. Overall, the BET specific surface area, micropore surface area, and micropore volume positively contribute to the CO_2_ adsorption of biochar. Conversely, pore width and O*/C are negatively correlated to the CO_2_ adsorption capacity. Moreover, the I_D_/I_G_ measured by Raman spectroscopy does not indicate a clear correlation. At present, researchers have overestimated the importance of micropores for CO_2_ adsorption. For example, Figures 11(b) and 11(d) both show that CS has an abundant microporous structure, but the adsorption performance is lower than some activated BC and WP biochars. In this case, the pore width and O*/C need to be combined to evaluate the CO_2_ adsorption mechanism of the biochar. The large pore width results in CO_2_ passing through the interior of the biochar without capture, thus reducing the capture capacity of the biochar. Besides, the O*/C ratio, which reflects the polarity and hydrophilicity of the biochar,^47^ is negatively correlated with CO_2_ adsorption, indicating that biochars with high aromatization and hydrophobicity are favorable for CO_2_ capture. Therefore, based on the above description, the mechanism of CO_2_ adsorption by biochar is not dependent on a single factor but rather on a synergistic effect of pore size, surface area, elemental content, and surface functional groups.

**Figure 12:**
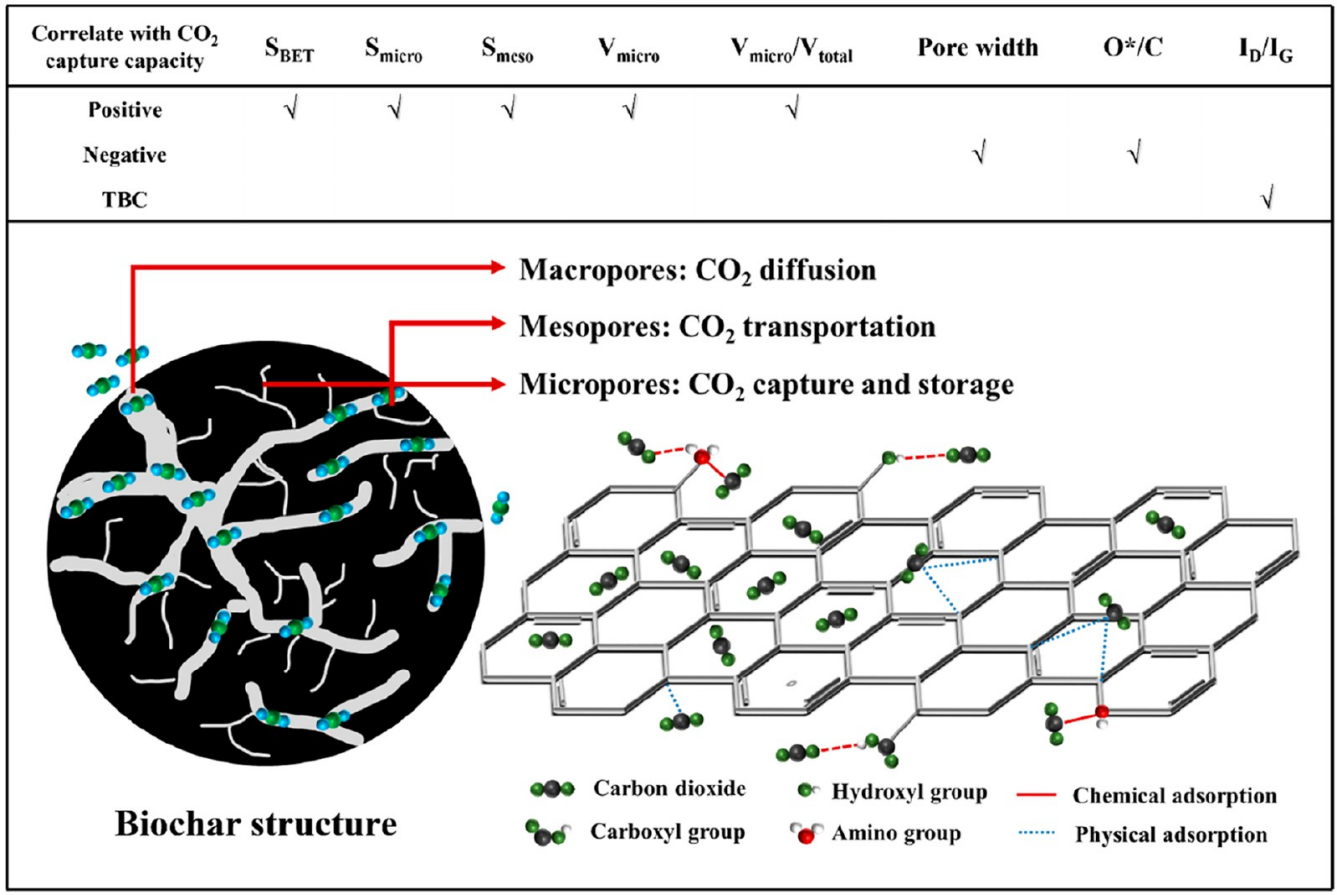
Potential mechanism of CO_2_ physisorption by a porous biochar material.

Overall, the physical adsorption of CO_2_ by biochar is related to its different properties. Figure 12 shows the positive (*S*_BET_, S_micro_, S_meso_, V_micro_, and V_micro_/V_total_) and negative (pore width, O*/C) correlation with CO_2_ capture properties. From the perspective of porosity, it normally decides the surface area and pore volume of adsorbents. The porous structure originates from the high-temperature pyrolysis of the precursors. The elimination of volatile substances and unstable elements from the precursors could generate the initial pore-size structure of the biochar. The role of pores of biochar for CO_2_ capture could be summarized: 1) macropores act primarily as diffusers, providing space for CO_2_ to come into complete contact with the biochar material;^48^ 2) mesopores act primarily as transporters, allowing CO_2_ to move freely within the mesoporous channels;^18^ and 3) micropores are the primary sites for CO_2_ adsorption and storage, allowing CO_2_ fixed inside the biochar under milder conditions. The microporosity shows intense CO_2_ adsorption potentials because overlapping the potential fields from the adjacent biochar layers increases the CO_2_ uptake capacity.^49^ Typically, both the specific surface area and pore size structure are correlated, with greater specific surface area generated from the rich porosity. Therefore, a higher specific surface area is more favorable for CO_2_ adsorption since a larger specific surface area provides sufficient adsorption sites.^1^ However, an increase in the specific surface area of biochar cannot be achieved without the activation process of the biochar material. The specific surface area can be enhanced with increasing activation time, temperature, and activator concentration. However, it is demonstrated that excessive activation leads to structural collapse of the biochar material, which results in a lower peak specific surface area and destruction of the pore size structure.^18,50^ As shown in Figure 12, the biochar with a large pore width demonstrates inactive adsorption ability.

Moreover, the elemental composition of biochar (reflected for the O*/C ratio in Figure 12) determines the chemical properties of its surface, such as acidity, hydrophobicity, and polarity, which are all related to the biochar’s ability to capture CO_2_.^51^ From the results of the correlation analysis, the O*/C ratio shows a negative effect on the CO_2_ capture of biochar. With these oxygen-containing functional groups, the hydrophilic and polar nature of the biochar surface cause water vapor to occupy the CO_2_ adsorption sites,^52^ reducing the amount of CO_2_ adsorbed. Thus, under the dryness CO_2_ adsorption conditions, the reduction in the O*/C ratio indicates the aromaticity of biochar and increases carbon fixation, which enhances the CO_2_ capture capacity.

Therefore, although some chemisorption (relying on functional groups, e.g., amino acids) is present in biochar, physical adsorption is still the primary means of CO_2_ capture. Furthermore, the correlation analysis shows that the prospects for carbon adsorption on biochar still need to be improved. High surface area, porous structures, and strongly hydrophobic and highly aromatized biochar would be promising CO_2_ adsorbents.

## 4 Conclusion

This work investigated the modification of five activation methods based on three types of lignocellulosic biochar: bamboo charcoal, wood pellet, and coconut shell. Meanwhile, various biochars were tested for CO_2_ capture performance using thermogravimetric analysis, a portable gas analyzer, and CO_2_ isothermal adsorption. Furthermore, the final correlation analysis was carried out using different characterization methods with porous structure analysis as the primary characterization measure. Acid and molten salt activation of biochar reduce CO_2_ adsorption. The physical adsorption of CO_2_ by biochar is mainly related to the internal structure. For example, the increase in the BET specific surface area, micropore surface area, mesopore surface area, and micropore volume favored the adsorption of CO_2_ by the biochar materials. Furthermore, the adsorption of CO_2_ by biochar is affected by a synergistic effect of the above physical properties. The O*/C ratio is negatively correlated with the CO_2_ capture capacity. It is suggested that a high ratio of O*/C could enhance the acidity of the adsorbent and result in low activity for capturing CO_2_. In addition, biochar activation and modification are both necessary to generate high-efficiency CO_2_ adsorbents. Biochar with a high C/N content, low oxygen content, high specific surface area, and adequate porous structure is preferred for effective CO_2_ capture.

## Supporting Information Available

Additional characterization results including ICP-OES, XRD analysis, biochar surface Zeta potential, and experimental results for 15% CO_2_ capture breakthrough, CO_2_ and N_2_ adsorption isotherm (PDF)
