# Supplementary material for: The Application of Biochar for CO2 Capture: Influence of Biochar Preparation and CO2 Capture Reactors
Source: Ind Eng Chem Res. 2023 Jun 29;62(42):17168–81. doi: 10.1021/acs.iecr.3c00445 (PMC10603783; doi:10.1021/acs.iecr.3c00445)
Supplement: Supplementary file 1 — ie3c00445_si_001.pdf [file ie3c00445_si_001.pdf]

Supporting information

The application of biochar for CO_2 capture: influence of biochar preparation and CO_2 capture reactor

Chen Zhang[b,1], Ying Ji[a,1], Chunchun Li[b], Yingrui Zhang[b], Shuzhuang Sun[b], Yikai Xu[b], Long Jiang[a]*, Chunfei Wu[b]*

[a]*Institute of Refrigeration and Cryogenics, Zhejiang University, Hangzhou. 310027, China*

[b]*School of Chemistry and Chemical Engineering, Queens University Belfast, Belfast, UK BT7 1NN*

[1]*These authors contributed equally and mainly to this study.*

Corresponding authors: Dr Chunfei Wu (Tel: +44 2890975573; E-mail: c.wu@qub.ac.uk); Dr Long Jiang (E-mail: jianglong@zju.edu.cn)

This supporting information file includes:

Supporting information Table S1. ICP-OES results of original biochars……………………………..……2

Supporting information Figure S1. X-ray diffraction patterns……………………………………….…..…2

Supporting information Figure S2. Zeta-potential………………………………………………...…….3

Supporting information Figure S3. 15% CO_2 capture breakthrough curve…..…………………………3

Supporting information Figure S4. CO_2 and N_2 adsorption isotherms at 298K…………………………4

Supporting information Table S1. ICP-OES results of original biochars

Table S1 depicts the major metal and inorganic elemental content of the three biochar raw materials, which slightly affects the characterization results, such as XRD.

Table S1: ICP-OES results of original biochars

	**BC-Origin**	**WP-Origin**	**CS-Origin**
**Ca (mg kg^{-1})**	3680.2	24235.2	1037.5
**K (mg kg^{-1})**	10905.6	6998.3	16368.0
**P (mg kg^{-1})**	781.3	1208.1	737.4
**Si (mg kg^{-1})**	3451.5	1787.1	2571.8

Figure S1 shows the XRD pattern of the biochar, which contains a certain amount of Si inorganic signal due to the presence of elemental Si. There are also slight differences in the signals of the plots depending on the activation methods, e.g., the XRD results of the $ZnCl_2$-activated samples show a certain amount of Zn-containing oxides.

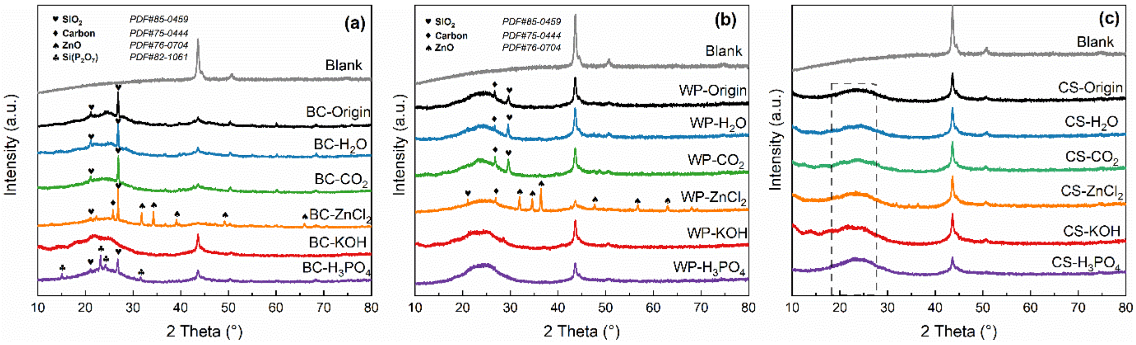

Figure S1: X-ray diffraction patterns of (a) bamboo charcoal biochars, (b) wood pellet biochars, and (c) coconut shell biochars.

Supporting information Figure S6. Zeta-potential.

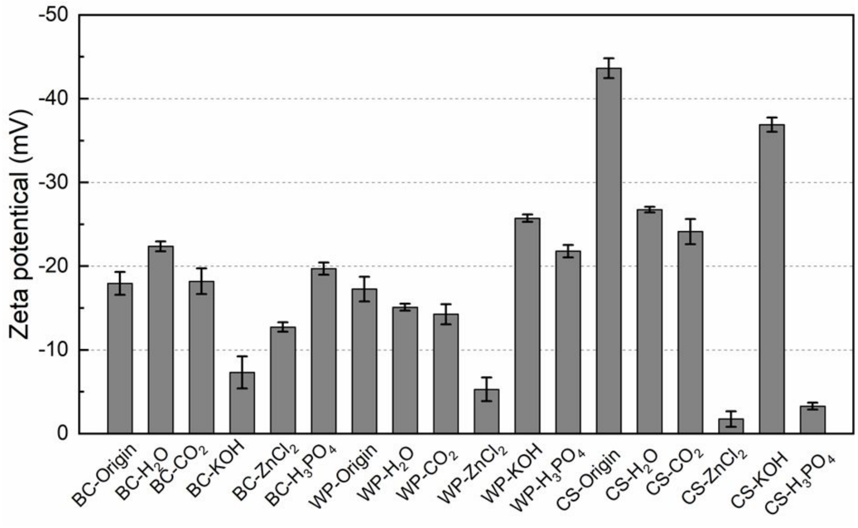

Figure S2: Zeta potentials of lignocellulosic biochar.

Supporting information Figure S3. 15% CO_2 capture breakthrough curve.

Figure S3 introduces the breakthrough curves for a variety of biochar adsorption through a fixed-bed reactor, and the curve for the blank test has been deducted.

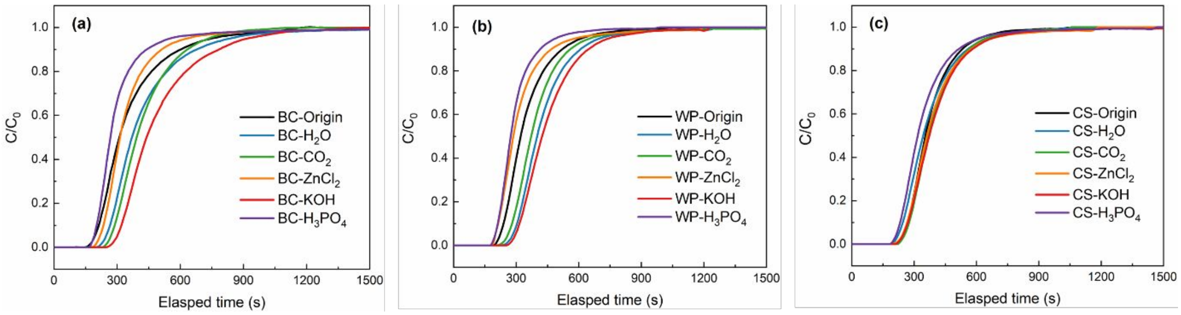

Figure S3: 15% CO_2 capture breakthrough curve of (a) bamboo charcoal biochars, (b) wood pellet biochars, and (c) coconut shell biochars.

Supporting information Figure S4. CO_2 and N_2 adsorption isotherms at 298K.

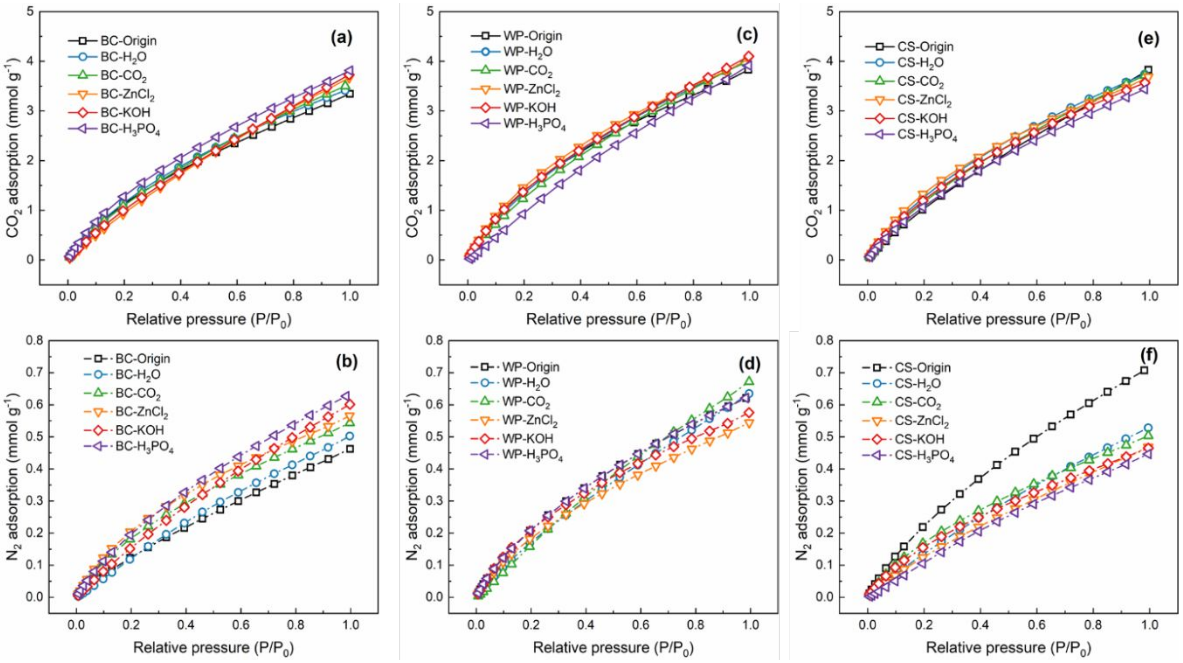

Figure S4. CO_2 and N_2 adsorption isotherms of (a)&(b) BC samples, WP samples (c)&(d) and CS samples (e)&(f) at 298K.
